# Marginal Accuracy of Ceramic Veneer Alloy Related to Different Alloy Fabrication Techniques, Ceramic Veneering Methods, Stages, and Sites of Fabrication

**DOI:** 10.1055/s-0044-1795079

**Published:** 2024-11-21

**Authors:** Sanephume Sripairojn, Niwut Juntavee, Apa Juntavee

**Affiliations:** 1 Division of Biomaterials and Prosthodontics Research, Faculty of Dentistry, Khon Kaen University, Khon Kaen, Thailand; 2 Department of Prosthodontics, Faculty of Dentistry, Khon Kaen University, Khon Kaen, Thailand; 3 Division of Pediatric Dentistry, Department of Preventive Dentistry, Faculty of Dentistry, Khon Kaen University, Khon Kaen, Thailand

**Keywords:** digital impression, marginal accuracy, metal ceramic, press-on ceramic, sintered metal

## Abstract

**Objectives:**

Fabrication processes affect accuracy of restoration. This study compared marginal accuracy of ceramic veneer metal upon different metal substructure fabrication techniques, ceramic veneering methods, stages, and sites of restoration.

**Material and Methods:**

A prepared premolar metal abutment was used to fabricate 96 metal substructures from 4 techniques: cast metal with traditionally impressed tooth (CmTt), cast metal with digitally milled wax (CmDw), sintered metal with digitally impressed tooth (SmDt), and sintered metal with digitally impressed stone model (SmDm). As-cast (A) substructures were degassed (D), opaqued (O), and contoured (C) with porcelain layering (Pl) or press-on (Pp) methods and glazed (G). Marginal fit was measured at A, D, O, C, and G stages, on buccal (Bu), lingual (Li), mesial (Me), and distal (Di) sites using silicone replica.

**Statistical Analysis:**

Analysis of variance and Bonferroni test were analyzed for significant differences of marginal fit upon different factors ( *α* = 0.05).

**Results:**

Significantly different accuracy was found upon metal substructures fabrication technique, veneering methods, stages, and sites of restoration ( *p* < 0.05). SmDt and SmDm revealed significantly better accuracy than CmTt and CmDw ( *p* < 0.05). Pp generated significantly better accuracy than Pl ( *p* < 0.05). Significant increasing inaccuracy was found at D stage ( *p* < 0.05). Me and Di sites exhibited larger inaccuracy than Bu and Li sites ( *p* < 0.05). However, marginal inaccuracy for all groups was under clinically acceptable marginal fit.

**Conclusion:**

Increasing marginal inaccuracies upon stages of fabrication were noticed, with highly observed at the proximal site. Sintered metal provided better accuracy than cast metal, while press-on veneering generated better accuracy than the layering method. Porcelain press-on sintered metal was suggested for fabrication restoration.

## Introduction

Metal ceramic has been effectively utilized as a restoration of privilege for restoring a damaged tooth because of the aesthetic ceramic property veneering on a strong metal substructure. 1 Construction of metal-ceramic restoration comprises of two steps; metal substructure fabrication and ceramic application. Predominated base metal alloys, either cobalt-chromium (Co-Cr) or nickel-chromium alloys, are frequently used due to their superior mechanical properties, biocompatibility, and lower cost compared with other alloys. The Co-Cr alloys are privileged for someone sensitive to nickel. 1 2 Nonnoble metal substructures are conventionally produced through the lost-wax technique, which is considered a more sensitive technique and inferior castability than noble metal alloys, owing to their extremely high melting temperature, and little ductile property. 3 4 Thus, some accumulating errors from the laboratory casting processes are unavoidable. Lately, computer-aided design and computer-aided manufacturing (CAD/CAM) technology has allowed metal substructures to be fabricated through a milling process by using data designed with CAD software. 5 6 7 The CAD/CAM systems are capable of producing higher accuracy and more reliability of restorations by milling hard alloy blank or presintered powder alloy blank. 8 However, milling hard alloy blank requires processing at the qualified grinding center which is costly. Milling the presintered powder alloy chalky-like blank offers an easier process to generate a green stage substructure and further sinters to accomplish the sintered metal substructure, which is less time-consuming and cost process. The mechanical properties of the sintered alloy were proved to be comparable to the hard milled alloy. 9 10 The CAD/CAM system is also capable of milling hard wax blank to fabricate a precise wax pattern coping for further transforming to cast metal substructure through the casting process. The accuracy of the sintered alloy substructure is probably related to the omission of waxing, investing, and casting processes in the conventional casting method. 11

At present, veneering ceramic to metal substructure can be performed either by conventional porcelain layering or porcelain pressed-on methods. The layering method demands proficiencies of dental technicians and needs many porcelain firings to accomplish aesthetic final restoration that may perhaps commence many inaccuracies during the fabrication process. 12 The press-on technique facilitates easier producing high-quality metal-ceramic restorations. A fully anatomical wax-up was accomplished on the substructure by pressing the melted ceramic onto the substructure. 13 This technique avoids multiple ceramic firings and results in achieving precise anatomical form and occlusal relationship of the restorations to the antagonist's tooth. Better distribution of crystalline phases in the glass matrix enables minimal ceramic shrinkage and better marginal accuracy. 13

Marginal accuracy plays an essential part in the long-term success of fixed prostheses. Improper marginal adaptation of restoration provokes microbacteria to pass through the gap, which potentially causes dental caries and periodontal disease that precedes failure. 14 Precise restorative margin diminishes the possibility of diseases related to tooth abutment plus prolongs restoration longevity. 14 The distortion of the metal-ceramic margin probably increased after ceramic veneering, firings, and glazing, which may perhaps prevent the marginal adaptation of restorations to abutment. 3 15 16 However, dimensional changes mostly occur during metal substructure cooling. 3 17 This is possibly related to the framework design, fabrication technique, type of alloy, shrinkage of ceramic, and the coefficient of thermal expansion (CTE) difference between alloy and ceramic. 1 2 3 4 7 13 18 Some studies reported the porcelain firing cycle effect on marginal accuracy, 19 20 while the others did not. 13 15 Usually, the clinically acceptable marginal discrepancy should be less than 120 μm. 21 Yet, an approximately 25 to 50 μm space between abutment and restoration is required for even luting cement thickness together with the feasibility of proper seating restoration upon cementation. 22 23 Moreover, a luting agent tends to be exposed to oral saliva and gradually dissolves upon a larger cement space. 24 Improper marginal discrepancy can produce stress concentrations that result in reducing the strength, and retention of the restorations. 15 24 25

The impact of different techniques of metal-ceramic fabrication on marginal accuracy has been a crucial consideration for dental practitioners. 1 12 25 Superior marginal accuracy is required to ensure clinical reliability. Both the sintered metal substructure fabrication technique and the press-on ceramic veneering technique are promising future of metal-ceramic restoration. 6 7 12 Nevertheless, there was limited information on the marginal accuracy of ceramic veneer sintered alloy related to ceramic veneering methods, as well as limited connecting to practicing processes. 12 Hence, this *in vitro* study intended to assess the marginal accuracy of ceramic veneer metal crowns fabricated from four metal substructure fabrication techniques that were veneered with two ceramic veneering methods, along five stages of restoration fabrication, and at four sites of restorative margin, with the research design relevant to clinical practice processes. The null hypothesis of this investigation was that the marginal accuracy of ceramic veneer metal crown not being significantly affected by the difference in metal substructure fabrication techniques, ceramic veneering methods, stages of restoration fabrication, sites of restorative margin, and their interactions. Conventional metal ceramic crowns based on traditional fabricated cast metal veneered with conventional porcelain layering served as a control group.

## Materials and Methods

The estimated sample size estimation was computed by G*power 3.1 software (Heinrich-Heine-Universität, Düsseldorf, Germany) using the statistical values of Pettenò et al's publication 26 at the power of test = 0.9 and *α* error = 0.05 as shown in Eq. 1 .



where *Z _α_* is the normal standard deviation = 1.96 ( *α* error = 0.05), *Z _β_* is the normal standard deviation = 1.28 ( *β* error = 0.1), µ _1_ –µ _2_ is the differences of mean between tested groups = 5, and *s* is the standard deviation ( *s*_1_ = 5, *s*_2_ = 2).

The calculated sample size was 12 specimens/group used for this investigation.

### Fabrication Master Model

**Fig. 1:**
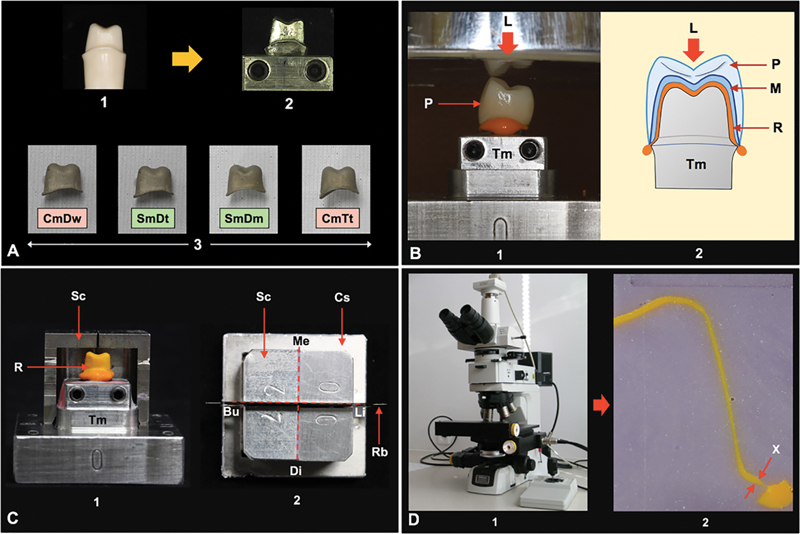
( **A** ) A typodont maxillary first premolar was prepared (1), replicated to a cast metal tooth, positioned on the metal base model (2) using for (3) fabrication of four types of metal substructures including cast metal with traditionally impressed tooth [CmTt], sintered metal with digitally impressed tooth [SmDt], sintered metal with digitally impressed stone model [SmDm], and cast metal with digitally milled wax [CmDw]. ( **B** ) The intaglio surface of the porcelain (P) veneer metal (M) crown was filled with a light viscosity silicone impression material (1), placed onto the metal tooth abutment (Tm), and constantly loaded (L) in apical direction (2). ( **C** ) The silicone replica (R) was picked up with regular viscosity silicone impression material using split mold metal cap (Sc) and cap stabilizer (Cs) (1) for further sectioning in mesial-distal (Me-Di) and buccal-lingual (Bu-Li) directions using razor blades (Rb) (2). ( **D** ) A polarizing microscope (1) was used to determine marginal discrepancy (X) using Image J software (2).

**Fig. 2:**
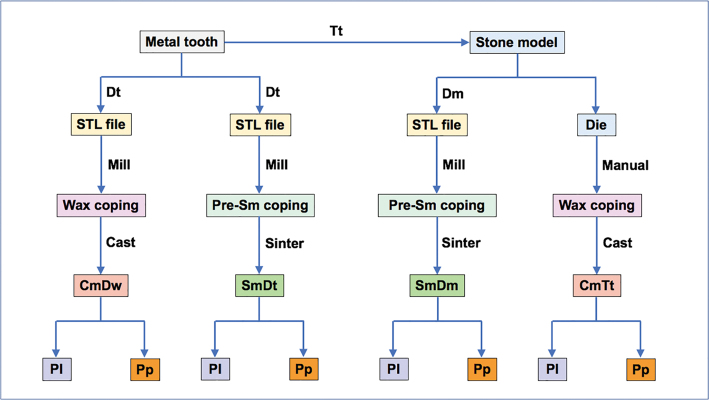
Prepared metal tooth was traditionally impressed (Tt) to fabricate 48 stone models. Twenty-four Standard Tessellation Language (STL) files were generated from digitally impressed metal tooth (Dt), designed, milled wax (Dw) copings to fabricate cast copings (Cm) upon the CmDw technique. Twenty-four STL files were generated by Dt, designed, milled presintered copings, and sintered to derive sintered metal (Sm) copings upon the SmDt technique. Twenty-four STL files were generated by digitally impressed stone models (Dm), designed, milled presintered copings, and sintered to reach for sintered metal (Sm) copings using the SmDm technique. Twenty-four stone dies were manually carved for wax copings to fabricate cast metal (Cm) copings using the CmTt technique. All metal copings were randomly veneered with ceramic by either porcelain layering (Pl) or porcelain press-on (Pp) technique.

A typodont maxillary first premolar (Frasaco, Tettnang, Germany) was prepared for the metal-ceramic crown with diamond rotary instruments (Khon Kaen University Preparation Kit 1918, Jota, Ruthi, Switzerland) and high-speed handpiece (KaVo, Biberarch, Germany). The preparation was designed for 1.5 mm identical axial and occlusal reduction, a 1.2-mm smooth continuous chamfer finishing line located 0.5 mm above the cementoenamel junction with a round internal line angle, and 10 degrees total occlusal convergence angle ( Fig. 1A-1 ). The prepared tooth was duplicated with polyvinyl siloxane (PVS) impression material (Silagum, DMG, Hamburg, Germany) and poured with pattern resin (Duralay, Reliance, Alsip, Illinois, United States). The resin pattern was invested in the casting ring using phosphate-bonded investment (Ceramvest Hi-Speed; Protechno, Girona, Spain) to transform to cast metal through the process of loss wax technique. The investment mold was burnt-out in a furnace (EWL-5645, Kavo) and cast with predominated base metal alloy (d.SIGN 30, Ivoclar Vivadent, Schaan, Liechtenstein) using a centrifuged casting apparatus (Fornax, Bego, Bremen, Germany). The metal casting specimens were divested and sandblasted with 110 μm aluminum oxides (Al _2_ O _3_ ) abrasive (Korox, Bego) in a sandblasting machine (Vario, Renfert, Hilzingen, Germany) to remove the remaining residue. The sprues were cut with Al _2_ O _3_ cutting disk (Shofu, Kyoto, Japan), and the cast was finished surface with Dura-Green stone burs (Shofu), polished with gold polishing kit (Shofu), and ultrasonically cleaned with the distilled water for 15 minutes in a cleansing machine (Vitasonic, Vita Zahnfabrik, Bad Sackingen, Germany) to remove the remaining Al _2_ O _3_ particles and cleaned with streaming machine (Touchsteam, Kerr, Brea, California, United States) to eliminate the grease residues. The metal tooth abutment was positioned at the central portion of the metal base (width × length × thick = 3 × 4 × 3 cm) and used as a master model in this investigation ( Fig. 1A-2 ).

### Fabrication of Metal Substructure

Ninety-six (96) metal substructures were designed for 0.3 mm thickness with narrow metal collars all around the margin and consistently fabricated from four different techniques according to the diagram shown in Fig. 2 . All laboratory processes for the fabrication of ceramic veneered metal were performed by one well-qualified dental technician who is capable of performing both digital and conventional fixed prosthesis, followed the standardized protocols, and conducted in the professional dental institute under the supervision of the Professor of Prosthodontics belonging to the institute. The metal substructure fabrication techniques were described as follows.

#### Cast Metal (Cm) Constructed from Traditionally Impressed Tooth (Tt) (CmTt Technique)

Traditional impressions of the prepared metal tooth (Tt) were performed by using light viscosity and putty-soft PVS impression material (Silagum, DMG) using a double-mixed technique with a customized autopolymerizable resin (Formatray, Kerr) trays and then poured the impression with type IV dental stone (Vel-Mix, Kerr) to fabricate stone models and dies. The hardening solution (Bredent, Senden, Germany) was applied to the stone casts. Two layers of red color die spacer (Durolan, DFS, Riedenburg, Germany) were applied on the stone dies with 0.5 mm clearance from the finishing line of the abutments to reach an equivalent thickness of 20 μm and let dry for 60 seconds. The wax pattern copings with standardized control shape and thickness were manually fabricated by a wax-dipping method using the blue inlay casting wax (Kerr) and transformed to cast metal (Cm) substructures through the loss wax technique as previously described to derive 24 CmTt copings.

#### Cast Metal (Cm) Constructed from Digitally Milled Wax (Dw) (CmDw Technique)

Digital impressions of the prepared metal tooth were obtained by scanning with a confocal microscopy-based intraoral scanner (TRIOS 5, 3Shape A/S, Copenhagen, Denmark) to produce Standard Tessellation Language (STL) files to be used in designing substructures with the CAD software (Ceramill Mind v2.7.05, Amann Girrbach, Koblach, Austria) by setting marginal discrepancy for 0 mm, thickness of 0.3 mm, and 20 µm simulated die spacer starting 0.5 mm away from the finishing line of the prepared abutment. The intended data were assigned to a 5-axis CAM-milling instrument (Ceramill Motion 2; Amann Girrbach) for milling 24 digitally milled wax (Dw) copings from the hard wax blank (Ceramill Wax, Amann Girrbach) for further fabrication of 24 CmDw copings by mean of loss wax technique as previously described.

#### Sintered Metal (Sm) Constructed from Digitally Impressed Tooth (Dt) (SmDt Technique)

Digital impressions of the prepared metal tooth (Dt) were obtained by scanning with an intraoral scanner (TRIOS 5, 3Shape A/S) to produce STL files, and used for designing substructures with the CAD software (Ceramill Mind v2.7.05, Amann Girrbach), with the previous identical design, but setting 15% larger than required. The proposed data were assigned to mill the presintered Co-Cr alloy blank (Ceramill Sintron, Amann Girrbach) in a CAM-milling device (Ceramill Motion 2; Amann Girrbach) to produce presintered metal copings and further sintering at 1,300°C for 6 hours in an argon gas chamber of the furnace (Ceramill Argotherm-2, Amann Girrbach) to derive 24 SmDt copings.

#### Sintered Metal (Sm) Constructed from Digitally Impressed Model (Dm) (SmDm Technique)

Conventional stone models and dies were fabricated as previously described. The digital impressions of the stone models (Dm) were obtained by scanning with a cast scanning machine (Ceramill Map-400; Amann Girrbach) to produce STL files and used for designing substructures with the CAD-software (Ceramill Mind v2.7.05, Amann Girrbach), with the previous identical design, but setting 15% larger than required. The intended data were assigned to a CAM-milling device (Ceramill Motion 2; Amann Girrbach) to produce presintered metal copings from Co-Cr presintered alloy blank and further sintered in a furnace as previously described to derive 24 SmDm copings.

### Surface Preparation of Metal Substructure

All metal substructures were prepared on the surface by grinding with stone bur (Coral stones, Shofu) in a unique path at 20,000 revolutions per minute speed and then blasted with 110 μm Al _2_ O _3_ (Korox, Bego) using 2 bar pressure for 10 seconds in a sandblaster unit (Vario, Renfert) with placing the tip 10 mm away from the substructure. The samples were cleaned ultrasonically in distilled water for 30 minutes and then steam-cleaned for 15 seconds. All metal substructures were heat treated in the furnace (Programat, Ivoclar Vivadent) to oxidize the metal surface by degassing process (D) as following the manufacturer's instruction ( Table 1 ).

**Table 1:** (A) Firing parameters for traditional layering ceramic for and press-on ceramic and (B) list of word/phrase abbreviations

A. Firing parameters for traditional layering ceramic for and press-on ceramic								
Ceramic	Sintering stages	Temperature (°C)		Closing time (min)	Heat rate (°C/min)	Holding time (min)	Vacuum (°C)	
		Start	Firing				On	Off
Traditional layering (IPS InLine)	Oxidized firing	403	980	4	80	1	450	979
	Opaque firing	403	930	6	100	2	450	929
	Dentine firing	403	910	4	60	1	450	909
	Glaze firing	403	830	6	60	1	450	829
Press-on (IPS InLine PoM)	Oxidized firing	403	980	4	80	1	450	979
	Opaque firing	403	930	6	100	2	450	929
	Pressed firing	700	960	4	60	1	500	960
	Touch up firing	403	840	4	60	1	450	839
	Glaze firing	403	710	6	60	1	450	709
**B. List of abbreviations (Abv.)**								
Word/Phrase		Abv.	Word/Phrase		Abv.	Word/Phrase		Abv.
Cast metal		Cm	As-cast		A	Buccal		Bu
Sintered metal		Sm	Degass		D	Lingual		Li
Traditional impressed tooth		Tr	Opaque		O	Mesial		Me
Digitally impressed tooth		Dt	Contour		C	Distal		Di
Digitally milled wax		Dw	Glaze		G	Razor blade		Rb
Cast metal with traditional impressed tooth		CmTt	Standard Tessellation Language		STL	Nickel-Chromium		Ni-Cr
Cast metal with digitally milled wax		CmDw	Silicone replica		R	Cobalt-Chromium		Co-Cr
Sintered metal with digitally impressed tooth		SmDt	Split mold metal cap		Sc	Porcelain layering		Pl
Sintered metal with digitally impressed stone		SmDm	Cap stabilizer		Cs	Porcelain press-on		Pp

### Opaque Porcelain Application

The A3 paste opaque porcelain (IPS Inline, Ivoclar Vivadent) was smeared to all metal copings and fired twice as stated by the company's firing instruction ( Table 1 ) in the ceramic furnace (Programat, Ivoclar Vivadent) to derive the 0.1-mm thickness of opaque layer. The first opaque porcelain layer was sparsely smeared and fired. The second opaque porcelain layer was completely applied over the first layer and fired to achieve an eggshell appearance surface. Each type of metal substructure was randomly allocated into two subgroups to be veneered with porcelain either by conventional porcelain layering (Pl) or porcelain press-on (Pp) technique ( Fig. 2 ).

### Conventional Porcelain Layering Technique

The samples were veneered with porcelain with the Pl technique to the desired shape and thickness. A creamy uniformity of A3 dentine porcelain (IPS InLine, Ivoclar Vivadent) was spread over the opaque surface, condensed with an ultrasonic condensing machine (Ceramosonic, Unitek, Osaka, Japan) to generate crown contour, and fired in the ceramic furnace (Programat, Ivoclar Vivadent) following the company's instructions ( Table 1 ). The dentine porcelain was permitted for two applications to derive the final contour of 1.2 mm thickness, using a jig to control an anatomical crown contour, and finally glazed.

### Porcelain Pressed-On Technique

The samples were coated with the modeling wax (Geo classic, Renfert) for 1.2 mm thickness to fabricate the anatomical crown contour, attached the sprue to the wax portion, invested in the silicone investing ring, using a phosphate-bonded investment (IPS PressVEST Speed; Ivoclar Vivadent) and burned out in the furnace according to the manufacturer's instruction. Once the burned-out process is completed, the investment mold was relocated to a pressed oven (EP 500, Ivoclar Vivadent) for the porcelain pressing process (Pp), using A3 porcelain ingots (IPS InLine PoM, Ivoclar Vivadent). After the pressed process was completed, the divestment process was performed by blasting with 110 µm Al _2_ O _3_ abrasive (Korox, Bego) with a pen blaster (Vario, Renfert) by locating the blasting tip at 20 mm far from the porcelain crown, with 4 bars pressure, for 10 seconds. Once the pressed ceramic became visible, the blasting pressure was reduced to 1.5 bars, and gently blasted the ceramic surface. A diamond separating disk (Kerr) was used to separate the sprues from the crown surface. Then, the samples were finished, polished, and glazed.

### Evaluation of Marginal Accuracy

The silicone replica method was used to assess the marginal accuracy of restoration to the metal tooth abutment (Tm) on the master metal model. 1 27 The marginal fit was measured at each stage of restorative fabrication including as-cast metal coping (A), degassing (D), opaquing (O), body contouring (C), and glazing (G). The intaglio surface of the metal substructure was inspected under a widefield zoom stereomicroscope (Carl Zeiss, Oberkochen, Germany) to confirm free of residue porcelain particles before evaluating the marginal gap at different stages of ceramic veneering. The intaglio surface of the restoration was covered with a light viscosity PVS material (Silagum, DMG), positioned on the Tm, together with constantly loaded for 50 N in the vertical direction until the PVS material completely polymerized ( Fig. 1B ). 28 Upon removal of the restoration from the Tm, a skinny layer of silicone replica (R) was left adhering on the Tm, representing the discrepancy between restoration and Tm ( Fig. 1C-1 ). A regular viscosity PVS material (Reprosil, Dentsply Sirona, Charlotte, North Carolina, United States) was applied into the split mold metal cap (Sc) and seated on the master metal model by keeping in place the cap stabilizer (Cs) to pick up the replica and further injected a regular viscosity PVS material (Reprosil, Dentsply Sirona) to the internal surface to stabilize the R as a sandwiching method. The R was longitudinally sectioned by two cuts using the superthin razor blades (Gillette, Boston, Massachusetts, United States) through the center of the replica in buccal (Bu)–lingual (Li) and mesial (Me)–distal (Di) directions ( Fig. 1C-2 ), leading to four sections of silicone replica used for measuring the marginal gap at Bu, Li, Me, and Di sites, yielding eight measurements for each stage of fabrication by using the polarized light microscope (Eclipse LV100pol, Nikon, Melville, New York, United States) ( Fig. 1D-1 ). The thickness of the silicone replica (R, yellow color) between the silicone (violet color) representing the edge of the metal coping and the silicone (violet color) representing the cavosurface margin of tooth abutment was measured under the microscope at ×30 magnification, analyzed by the Image J software (U.S. National Institute of Health, Bethesda, Maryland, United States), and was defined as a marginal discrepancy ( Fig. 1D-2 ). 2

### Statistical Analysis

The marginal gap data were analyzed with statistics software (SPSS/PC V-26, IBM, Armonk, New York, United States). Once the data were examined for the normal distribution using the Shapiro–Wilk test and homogeneity of the variances using the Levene's test, a comparison of the measured adjustments was analyzed using multifactorial analysis of variance (ANOVA) repeated measurement to conclude the statistically significant difference of marginal accuracy of the metal-ceramic restorations upon different metal substructure fabrication techniques, ceramic veneering methods, stages of fabrication, and sites of restorative margin. Post hoc Bonferroni multiple comparisons were applied to justify differences among factors at a 95% level of confidence.

## Results

**Fig. 3:**
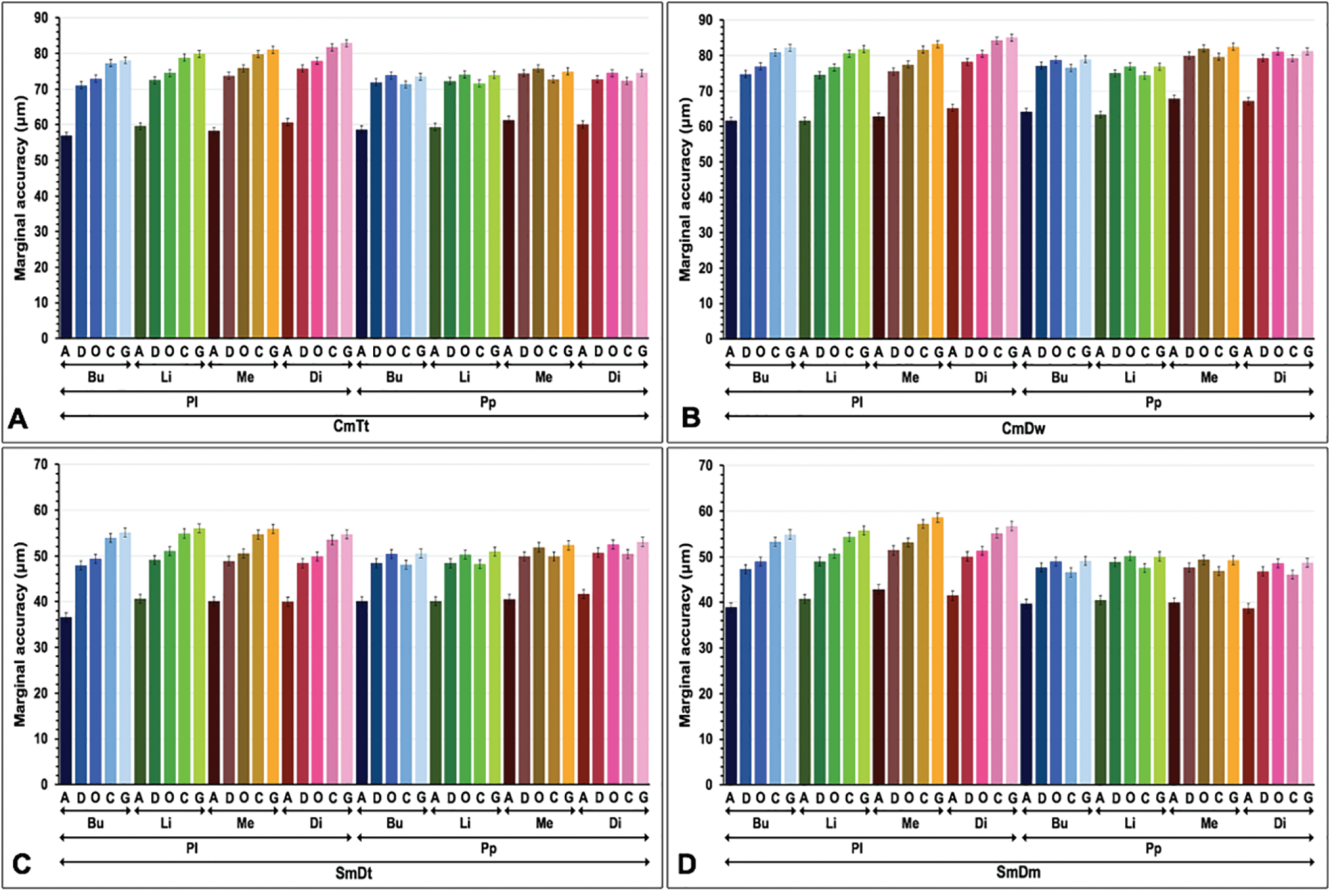
Mean marginal accuracy at different sites (buccal: Bu; lingual: Li; mesial: Me; distal: Di) of ceramic veneered ( **A** ) cast metal constructed from traditional loss wax (CmTt), ( **B** ) cast metal of the digitally impressed wax (CmDw), ( **C** ) sintered metal of digitally impressed tooth (SmDt), and ( **D** ) sintered metal of digitally impressed model (SmDm) techniques, with either porcelain layering (Pl) or porcelain press-on (Pp) at as-cast (A), degass (D), opaquing (O), body contouring (C), and glazing (G) stages.

The means and standard deviations of marginal accuracy for ceramic veneer metal restorations related to different metal substructure fabrication techniques, stages of restoration fabrication, methods of ceramic veneering, and sites of restorative margin were presented ( Table 2 and Fig. 3 ). Multifactorial ANOVA confirmed for significantly different marginal accuracy of restoration upon various metal substructure fabrication techniques, stages of restorative fabrication, methods of ceramic veneering, and sites of restorative margin ( *p* < 0.05). When interacting factors were taken into account, significant differences were detected between the metal substructure fabrication techniques and the stages of fabrication, between the metal substructure fabrication techniques and the methods of ceramic veneering, and together between the stages of fabrication and the methods of ceramic veneering ( *p* < 0.05). However, no significant differences in marginal accuracy were detected between the metal substructure fabrication techniques and the sites of restorative margin, between the methods of ceramic veneering and the sites of restorative margin, and among three and four factors interaction ( *p* > 0.05) ( Table 3 ).

**Table 2:** Mean and standard deviation (SD) of marginal accuracy (μm) at buccal (Bu), lingual (Li), mesial (Me), and distal (Di) sites of ceramic veneer cast (Cm) and sintered (Sm) metals constructed from traditional (Tt), digitally impressed tooth (Dt), digitally impressed model (Dm), and digitally milled wax (Dw) techniques, veneered with either porcelain layering (Pl) or porcelain press-on (Pp) method at the stages of as-cast (A), degassing (D), opaquing (O), body contouring (C), and glazing (G)

Substructure	Ceramic	Site	*n*	Stages of fabrication (mean ± SD)				
				A	D	O	C	G
CmTt	Pl	Bu	12	56.92 ± 7.66	71.08 ± 7.20	72.92 ± 8.10	77.25 ± 8.27	78.08 ± 8.40
CmTt	Pl	Li	12	53.58 ± 10.09	72.50 ± 9.42	74.5 ± 10.23	78.83 ± 10.07	79.92 ± 10.08
CmTt	Pl	Me	12	58.25 ± 8.17	73.75 ± 9.35	75.83 ± 9.76	79.83 ± 9.46	81.08 ± 9.67
CmTt	Pl	Di	12	60.67 ± 9.20	75.75 ± 9.58	77.92 ± 9.72	81.75 ± 9.73	82.92 ± 10.07
CmTt	Pp	Bu	12	58.67 ± 8.61	71.92 ± 8.71	73.83 ± 9.05	71.33 ± 8.10	73.50 ± 9.59
CmTt	Pp	Li	12	59.33 ± 7.94	72.25 ± 7.89	74.08 ± 8.07	71.58 ± 8.59	73.92 ± 8.66
CmTt	Pp	Me	12	61.33 ± 8.90	74.42 ± 8.07	75.75 ± 8.47	72.75 ± 8.42	74.92 ± 8.37
CmTt	Pp	Di	12	60.08 ± 10.08	72.75 ± 9.71	74.50 ± 9.44	72.33 ± 9.44	74.50 ± 9.66
CmDw	Pl	Bu	12	61.58 ± 7.28	74.75 ± 7.65	76.92 ± 8.03	80.92 ± 8.02	82.25 ± 8.11
CmDw	Pl	Li	12	61.58 ± 5.99	74.5 ± 6.42	76.67 ± 6.83	80.58 ± 7.05	81.83 ± 7.20
CmDw	Pl	Me	12	62.75 ± 7.38	75.5 ± 7.73	77.42 ± 8.32	81.67 ± 8.35	83.25 ± 8.95
CmDw	Pl	Di	12	65.25 ± 6.23	78.17 ± 6.45	80.50 ± 7.22	84.25 ± 7.12	85.08 ± 6.97
CmDw	Pp	Bu	12	64.17 ± 8.72	77.08 ± 8.92	78.83 ± 9.44	76.50 ± 8.99	79.00 ± 9.32
CmDw	Pp	Li	12	63.33 ± 6.20	75.00 ± 6.35	76.92 ± 6.54	74.33 ± 6.71	76.83 ± 6.87
CmDw	Pp	Me	12	67.83 ± 9.20	80.00 ± 8.70	82.00 ± 9.16	79.67 ± 8.90	82.50 ± 8.74
CmDw	Pp	Di	12	67.17 ± 6.65	79.33 ± 6.40	81.17 ± 6.41	79.25 ± 6.55	81.25 ± 6.93
SmDt	Pl	Bu	12	36.58 ± 9.68	47.92 ± 6.27	49.42 ± 6.52	53.92 ± 6.23	55.08 ± 7.06
SmDt	Pl	Li	12	40.67 ± 4.94	49.17 ± 5.18	51.08 ± 5.63	54.92 ± 5.73	56.00 ± 5.95
SmDt	Pl	Me	12	40.08 ± 7.10	48.92 ± 7.23	50.58 ± 7.37	54.67 ± 7.75	55.92 ± 7.53
SmDt	Pl	Di	12	40.00 ± 6.89	48.42 ± 7.08	49.92 ± 6.93	53.50 ± 7.08	54.75 ± 7.35
SmDt	Pp	Bu	12	40.08 ± 7.60	48.42 ± 7.87	50.42 ± 7.90	48.08 ± 8.09	50.58 ± 7.95
SmDt	Pp	Li	12	40.17 ± 6.35	48.42 ± 6.52	50.33 ± 6.58	48.25 ± 7.02	50.92 ± 7.37
SmDt	Pp	Me	12	40.58 ± 5.76	49.92 ± 5.23	51.92 ± 5.07	49.92 ± 5.50	52.33 ± 5.91
SmDt	Pp	Di	12	41.67 ± 5.37	50.75 ± 5.48	52.50 ± 5.28	50.42 ± 5.58	53.08 ± 5.85
SmDm	Pl	Bu	12	38.92 ± 6.60	47.33 ± 6.44	49.00 ± 6.82	53.33 ± 6.96	54.92 ± 7.13
SmDm	Pl	Li	12	40.75 ± 4.54	49.00 ± 4.20	50.67 ± 4.25	54.33 ± 3.96	55.75 ± 4.33
SmDm	Pl	Me	12	42.92 ± 5.38	51.42 ± 6.47	53.17 ± 6.82	57.25 ± 6.54	58.58 ± 6.82
SmDm	Pl	Di	12	41.5 ± 5.82	50.08 ± 5.76	51.33 ± 6.02	55.17 ± 6.10	56.75 ± 6.48
SmDm	Pp	Bu	12	39.75 ± 5.43	47.67 ± 5.28	49.00 ± 5.51	46.58 ± 5.74	49.08 ± 5.81
SmDm	Pp	Li	12	40.5 ± 5.58	48.83 ± 6.01	50.17 ± 6.28	47.58 ± 6.42	50.08 ± 6.72
SmDm	Pp	Me	12	40.00 ± 8.25	47.67 ± 8.16	49.33 ± 8.25	46.92 ± 8.66	49.25 ± 8.16
SmDm	Pp	Di	12	38.75 ± 6.02	46.83 ± 6.69	48.58 ± 7.01	46.08 ± 6.79	48.75 ± 6.61

**Table 3:** Analysis of variance (ANOVA) of marginal accuracy at different sites of ceramic veneer metal substructures constructed from different techniques with different veneering methods upon various stages of fabrication

Source	SS	df	MS	*F*	*p*
Corrected model	381503.731	159	2399.395	42.526	0.001
Intercept	7253804.269	1	7253804.269	128564.334	0.001
Substructure	312204.706	3	104068.235	1844.475	0.001
Stage	56733.153	4	14183.288	251.381	0.001
Ceramic	1976.408	1	1976.408	35.029	0.001
Site	1460.727	3	486.909	8.630	0.001
Substructure * Ceramic	1065.050	3	355.017	6.292	0.001
Substructure * Site	731.381	9	81.265	1.440	0.165
Substructure * Stage	1762.643	12	146.887	2.603	0.002
Ceramic * Site	170.404	3	56.801	1.007	0.389
Ceramic * Stage	4411.857	4	1102.964	19.549	0.001
Site * Stage	25.351	12	2.113	0.037	1.000
Substructure * Ceramic * Site	782.088	9	86.899	1.540	0.128
Substructure * Ceramic * Stage	44.664	12	3.722	0.066	1.000
Substructure * Site * Stage	35.186	36	0.977	0.017	1.000
Ceramic * Site * Stage	18.872	12	1.573	0.028	1.000
Substructure * Ceramic * Site * Stage	81.241	36	2.257	0.040	1.000
Error	99302.000	1760	56.422		
Total	7734610.000	1920			
Corrected total	480805.731	1919			

Abbreviations: df, degree of freedom; *F* , *F* -ratio; MS, mean square; SS, sum of squares.

**Fig. 4:**
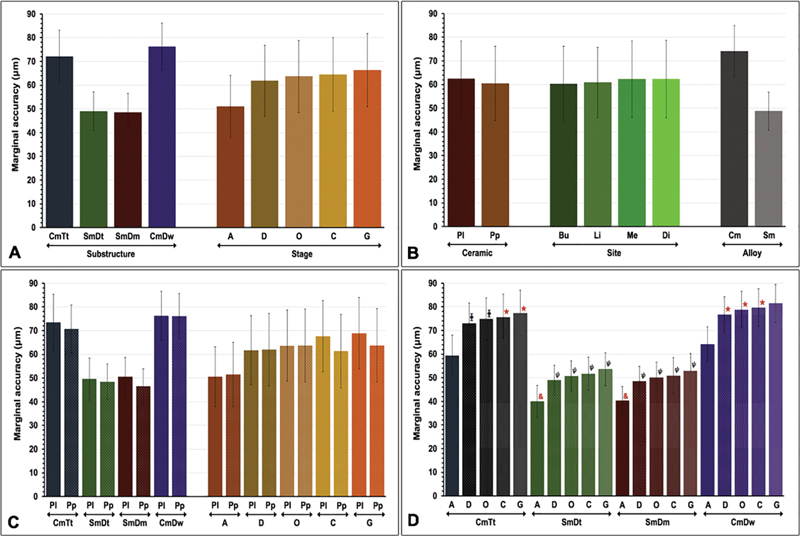
Marginal accuracy as a function of ( **A** ) substructure fabrication techniques (cast metal constructed from the traditional impressed tooth [CmTr], sintered metal constructed from the traditional impressed model [SmDt], sintered metal constructed from the digitally impressed model [SmDm], and cast metal constructed from digitally impressed wax [CmDw]), stages of fabrication (as-cast: A; degass: D; opaquing: O; body contouring: C; glazing: G), ( **B** ) ceramic veneering method (layering: Pl; press-on: Pp), sites (buccal: Bu; lingual: Li; mesial: Me; distal: Di), alloy types (cast metal; Cm, sinter metal; Sm), ( **C** ) interaction of substructure fabrication techniques with ceramic veneering methods, interaction of stages and ceramic veneering methods, and ( **D** ) interaction of substructure fabrication techniques and stages of fabrication (Note: same symbols above the bar graphs indicated no significant difference).

Post hoc Bonferroni multiple comparisons showed significant differences in marginal accuracy of restorations upon different factors tested ( Table 4 ). The marginal discrepancy of metal-ceramic restorations as a factor of the metal substructure fabrication techniques indicated that the CmDw (76.19 ± 9.86 µm) exhibited a significantly greater than that constructed from the CmTt (72.08 ± 11.15 µm), SmDm (49.01 ± 8.16 µm), and SmDt (48.59 ± 0.97 µm), respectively ( *p* < 0.05). However, no significantly different marginal discrepancy of restorations fabricated from both SmDm and SmDt techniques ( *p* > 0.05) ( Fig. 4A and Table 4A ). The marginal discrepancy of metal-ceramic restorations as a factor of stages of fabrication revealed a significant increase of marginal gap from A stage (50.98 ± 13.11 µm) to D (61.86 ± 14.95 µm), O (63.66 ± 15.20 µm), C (64.49 ± 15.54 µm), and G stage (66.33 ± 15.44 µm), respectively ( *p* < 0.05). Yet, no significant difference in the marginal discrepancy of restorative fabrication between the D and S stages was observed ( *p* > 0.05) ( Fig. 4A and Table 4B ). The marginal discrepancy of metal-ceramic restorations as a factor of ceramic veneering methods demonstrated a significantly larger marginal gap upon veneering with conventional layering (Pl) method (62.48 ± 15.86 µm) compared with the pressed-on veneering (Pp) method (60.45 ± 15.75 µm) ( *p* < 0.05) ( Fig. 4B and Table 4C ). The marginal discrepancy of metal-ceramic restorations as a factor of sites of restorative margin demonstrated a significantly larger marginal gap at the Me (62.30 ± 16.09 µm) and Di sites (62.34 ± 16.39 µm) compared with the Bu (60.34 ± 15.83 µm) and Li sites (60.89 ± 14.49 µm) ( *p* < 0.05). Nevertheless, no significant difference in marginal discrepancy of restoration between Me and Di sites as well as between Bu and Li sites was observed ( *p* > 0.05) ( Fig. 4B and Table 4D ). The statistics also indicated significantly better marginal accuracy upon the metal substructures fabricated from sintered metal (Sm, 48.80 ± 8.06 µm) compared with cast metal (Cm, 74.13 ± 10.72 µm) ( *p* < 0.05) ( Fig. 4B and Table 4E ). Post hoc Bonferroni multiple comparisons signified significant difference in marginal accuracy upon the different combinations of metal substructure fabrication techniques and methods of ceramic veneering ( *p* < 0.05), except between CmTtPl – CmDwPp groups, SmDtPl – SmDtPp – SmDmPl groups, and SmDtPp – SmDmPl – SmDmPp groups ( *p* > 0.05) ( Fig. 4C and Table 4F ). Post hoc Bonferroni multiple comparisons signified a significant difference of marginal accuracy upon the different combination of stages of restoration fabrication and methods of ceramic veneering ( *p* < 0.05), except between APl – APp groups, DPl – DPp – OPl – OPp – CPp – GPp groups, DPp – OPl – OPp – CPp – GPp groups, OPl – OPp – CPp – GPp groups, OPp – CPl – CPp – GPp groups, CPl – GPl– GPp groups, and CPp – GPp groups ( *p* > 0.05) ( Fig. 4C and Table 4G ). Post hoc Bonferroni multiple comparisons signified significant difference in marginal accuracy between the combination of metal substructure fabrication techniques and stages of restoration fabrication ( *p* < 0.05), except between CmTtD – CmTtO groups, CmTtC – CmTtG – CmDwD – CmDwO – CmDwC groups, SmDtD – SmDtO – SmDtC – SmDtG – SmDmD – SmDmO – SmDmC – SmDmG groups, and SmDtA – SmDmA groups ( *p* > 0.05) ( Fig. 4D ).

**Table 4:** Post hoc Bonferroni multiple comparisons of marginal accuracy of ceramic veneer alloy as a function of (A) substructures constructed from (cast: Cm; sintered: Sm) metal upon different techniques (traditional: Tt; digitally impressed tooth: Dt; digitally impressed model: Dm; and digitally milled wax: Dw), (B) stages of fabrication (as-cast: A; degass: D; opaquing: O; body contouring: C; glazing: G), (C) ceramic veneering methods (layering: Pl; press-on: Pp), (D) sites (buccal: Bu; lingual: Li; mesial: Me; distal: Di), (E) alloys, (F) interaction of substructures and veneering methods, and (G) interaction of veneering methods and stages of fabrication

**(A) Post hoc comparisons as a function of substructures**					**(B) Post hoc comparisons as a function of fabrication stages**					
**Substructure**	**CmTt**	**SmDt**	**SmDm**	**CmDw**	**Stage**	**M**	**D**	**O**	**C**	**G**
CmTt	1	0.001	0.001	0.001	M	1	0.001	0.001	0.001	0.001
SmDt		1	1	0.001	D		1	0.009	0.001	0.001
SmDm			1	0.001	O			1	1	0.001
CmDw				1	C				1	0.007
					G					1
**(C) Post hoc comparison of ceramics**			**(D) Post hoc comparisons as a function of sites**					**(E) Post hoc comparison of alloys**		
**Ceramic**	**Pl**	**Pp**	**Site**	**Bu**	**Li**	**Me**	**Di**	**Alloy**	**Cm**	**Sm**
Pl	1	0.001	Bu	1	1	0.001	0.001	Cm	1	0.001
Pp		1	Li		1	0.023	0.018	Sm		1
			Me			1	1			
			Di				1			
**(F) Post hoc comparison as an interaction of substructures and ceramic veneering methods**										
	**CmTtPl**	**CmTtPp**	**SmDtPl**	**SmDtPp**	**SmDmPl**	**SmDmPp**	**CmDwPl**	**CmDwPp**		
CmTtPl	1	0.030	0.001	0.001	0.001	0.001	0.027	0.053		
CmTtPp		1	0.001	0.001	0.001	0.001	0.001	0.001		
SmDtPl			1	1	1	0.012	0.001	0.001		
SmDtPp				1	1	0.787	0.001	0.001		
SmDmPl					1	0.001	0.001	0.001		
SmDmPp						1	0.001	0.001		
CmDwPl							1	1		
CmDwPp								1		
**(G) Post hoc comparisons of as an interaction of stages of fabrication and ceramic veneering methods**										
	**APl**	**APp**	**DPl**	**DPp**	**OPl**	**OPp**	**CPl**	**CPp**	**GPl**	**GPp**
APl	1	1	0.001	0.001	0.001	0.001	0.001	0.001	0.001	0.001
APp		1	0.001	0.001	0.001	0.001	0.001	0.001	0.001	0.001
DPl			1	1	1	1	0.005	1	0.001	1
DPp				1	1	1	0.008	1	0.001	1
OPl					1	1	0.350	1	0.022	1
OPp						1	0.420	1	0.028	1
CPl							1	0.001	1	0.483
CPp								1	0.001	1
GPl									1	0.033
GPp										1

## Discussion

Despite carefully prepared tooth abutments as well as a well-controlled process of restoration construction, impreciseness remains between the margins of the restorations and the prepared abutments, which predisposes to caries and periodontal disease of the tooth abutments. 14 The more precisely the margin of the restoration adapts to the finishing line of the prepared tooth, the smaller the marginal gap displays and the slighter the cement film is bared to oral fluid. This study confirmed the hypothesis that there were significant differences in marginal accuracy of ceramic veneer metal upon metal substructure fabrication techniques, stages of restorative fabrication, methods of ceramic veneering, sites of restorative margin, and their interaction between metal substructure fabrication techniques and stages of restoration fabrication, metal substructure fabrication techniques and methods of ceramic veneering, stages of restoration fabrication and methods of ceramic veneering, except for the interaction between metal substructure fabrication techniques and sites of restorative margin, methods of ceramic veneering and sites of restorative margin, and among three and four factors interaction, Hence, the null hypothesis was partially rejected. However, the marginal fit for all the studied groups was less than 120 μm, the generally accepted marginal misfit limit. 21 22

Regarding metal substructure fabrication techniques, the metal substructures fabricated either from SmDt or SmDm techniques exhibited better marginal accuracy than those fabricated either from CmTt or CmDw techniques, which were consistent with other studies. 1 3 5 18 This could be attributed to the conventional lost wax cast technique, which is a complex, sensitive, and nonreproducible method, and requires high skill dental technician to achieve a precise fit of restoration. The marginal discrepancy of metal substructures constructed from the CmTt technique is primarily associated with the dimensional accuracy of the conventional processes in the construction of the restoration including the traditional impression technique of the prepared tooth (Tt), dimensional accuracy of the stone model, and investing material together with the solidification shrinkage volume of metal upon casting that directly influenced the restorations fit. 28 The marginal discrepancy of the CmDw technique demonstrated a higher marginal discrepancy than the CmTt technique, which is probably related to the milled wax substructure that additionally increases error accumulation in this process. 20 This was supported by other studies that reported the marginal accuracy of the wax coping produced by hand carving was more precise than the wax coping produced by digitally milled wax. 6 9 The study indicated that metal substructure produced from sintered metal (Sm) provided better marginal accuracy than cast metal (Cm). This study was in agreement with other previous studies. 5 8 10 The metal substructures constructed from the digital impression procedure either from the intraoral scanner for making a digital impression of the prepared tooth or the laboratory scanner for performing a digital impression of the stone die of the stone model were both designed for the copings using a three-dimensional software program and then milled the presintered metal blank with a CAM milling machine. These techniques do not involve in the process of lost wax and for that reason, the stable dimensions seem to be achieved. Although fully sintered metal substructures must involve the shrinkage of powder metal upon sintering, the compensation was planned during CAD-designed substructures. It was considered to be the negligible fault inherent to every CAD-CAM system. 7 The digital impression process normally creates somewhat rounded borders related to the resolution for each scanner, which probably causes an early contact of restoration at the axial-occlusal edges and causes larger marginal inaccuracy. The process of digital impression-taking usually makes the overshooter peak around the edges of the target and causes a higher marginal inaccuracy. 11 This occurrence was expressed to exhibit to every single CAD-CAM that involves digital impressions. The cloud points gained from the digitally scanned process were converted into a continuously smooth surface depending on the proficiency of the designed software. This procedure can also steer to some impreciseness. However, the process of milling presintered metal blank is quite soft, seems easy, is not prone to establish marginal ditching, and has less stress accumulation on the presintered substructure. 1 8 18 The marginal accuracy of sintered metal substructures was comparable with either the presintered metal constructed from the STL file derived from digitally impressed tooth (Dt) or digitally impressed model (Dm). This is probably related to the preciseness of the prepared tooth and the stone die was comparable. The result was consistence with other studies that found no significant difference between the digital impression of the prepared tooth and the prepared stone die. 29 Furthermore, the study signified that the restoration constructed from sintered metal (Sm) revealed superior marginal accuracy than those constructed from cast metal (Cm) as supported by other studies. 5 8 10

As far as the stages of restoration fabrication were concerned, the accuracy of the restorative margin was affected by the sequential stages of fabrication. Before veneering with ceramic, the Sm substructure groups revealed superior marginal preciseness than the Cm substructure groups as supported by other studies. 1 2 A significant increase in marginal discrepancies of restorations upon the sequence of fabrication process from as-cast to degassing, opaquing, contouring, and glazing process, respectively, was evidenced, which corresponded with other studies. 1 12 16 19 The greatest increasing marginal discrepancies was found after degassing process. Metal substructures exposed to extremely high temperatures during the porcelain firing stage possibly produce dimensional distortion and finally decrease the preciseness of the restorative margin. The result of this study agreed with other studies that stated the greatest distortion of ceramic veneered metal occurs during the degassing stage. 12 16 19 This study used nonnoble Co-Cr alloy for fabricated metal substructure because of low cost, biocompatibility, resistance to corrosion, and stability in biological environments. However, the inherited disadvantage of nonnoble Co-Cr alloy is the thick oxide layer formation on the surface upon degassing process. This is probably related to the releasing of stresses upon the solidification process of alloy and the cold working process on the surface preparation before ceramic application. The degassing process occurs at an elevated temperature and could cause the grain growth of the deformed crystals and was postulated to cause greater marginal discrepancy of the degassed metal substructure as confirmed by other studies. 15 19 The increase in marginal inaccuracy after sequential ceramic sintering is generally influenced by numerous factors, for example, the ceramic firing shrinkage, the CTE difference of metal and ceramic, and the residual stresses generated from multiple firing processes. However, this study indicated that marginal discrepancies occurred after ceramic veneering, ceramic firing shrinkage as a causative factor in the marginal distortion, were not primary factors in distortion as in agreement with other studies. 1 20 23

The ceramic veneering technique could be associated with the marginal accuracy of metal-ceramic restorations, which causes less marginal distortion in the metal substructure veneered with pressable ceramics. The marginal accuracy of restoration veneered with pressed-on porcelain (Pp) was better than restoration veneered with conventional layering porcelain (Pl), which was supported by other studies. 13 20 24 This might be the effect of the number of ceramic firing cycles. The conventional ceramic veneering technique normally requires more firing cycles and higher skillfulness of dental technicians than the pressed-on techniques during the dentine porcelain contouring process. The press-on ceramic veneering technique requires full contour wax-up of the restoration on the substructure and then replaces it with pressed porcelain. This technique also eliminates technical errors from the porcelain firing process and multiple firings in conventional porcelain layering, thus reducing accumulated marginal discrepancies. 24 It was reported that increased marginal discrepancies form contamination of porcelain at the intaglio surface of metal substructure upon porcelain layering, while this event never occurred in the press-on technique. 20

The greater marginal discrepancy was exhibited at the proximal side than at the Bu and Li sides of the restoration. This is probably associated with the configuration of the restoration shrinkage through the processes of fabrication that are exhibited in three dimensions. The characteristics of firing shrinkage occur toward the center of the restoration and cause inaccuracy on the proximal site more than on other sites as other reports. 17 25 There are many techniques for evaluating marginal accuracy of restorations such as direct viewing, cement thickness measurement, cone-beam computed tomography, and silicone replica technique. 27 This study uses the silicone replica technique because it provides several advantages of measuring process without destroying the specimen, repeating the measurement, high reliability, and precision. 21 30 Since this study used the prepared premolar size metal tooth abutment that has the margin conforming with the geometry of the cementoenamel junction, thus the locations of the marginal accuracy were assigned at four sites on the buccal, lingual, mesial, and distal to eliminate the confounding effect from sectioning silicone replica upon other sites. Thus, it is suitable for the evaluation of marginal accuracy at different stages of fabrication in this study.

Since ceramic veneer sintered metal is comparatively new and rapidly utilized in clinical practice, there are several techniques to derive for final restorations. The information on the accuracy of restorative margin in this experiment founded on the systematized circumstances concerning study design, metal substructure fabrication techniques, ceramic veneering methods, method of evaluation, and experimental implementation provide realistically important scientific value for dentists in decision-making for dental reconstruction using digital approached ceramic veneer metal restoration in their dental practices. Nevertheless, upon the study's limitations, sintered metal substructures either fabricated from digitally impressed tooth or digitally impressed stone model, whether ceramic veneering by layering or press-on methods, provided better marginal accuracy than cast metal substructures either fabricated from traditional lost wax or digitally milled wax, even if ceramic veneering by layering or press-on technique. Yet, all techniques described in the study achieved satisfactorily precise marginal fit and were clinically acceptable for the fabrication of ceramic veneer metal restorations for oral reconstruction.

## Conclusion

This study confirmed that the marginal accuracy of ceramic veneer metal restorations was significantly affected by the different metal substructure fabrication techniques, ceramic veneering methods, sequential stages of restorative fabrications, and sites of restorations. Sintered metal substructures either fabricated from digitally impressed tooth or digitally impressed stone models achieved better marginal accuracy than cast metal substructures either fabricated from traditional lost wax or digitally milled wax. Porcelain press-on metal substructure generated less marginal distortion than the conventional porcelain layering method. A continued increase in marginal discrepancies of ceramic veneer metal through the sequential restorative fabrication processes was addressed, with the greatest increasing marginal distortion occurring during the degassing process. Higher marginal discrepancies were exhibited more on the proximal sites than on the other sites. Nevertheless, the ceramic veneered metal restoration fabricated in this study has shown clinically acceptable marginal accuracy. The study suggests fabricating metal-ceramic restoration with a sintered metal substructure and veneered with a porcelain press-on technique to derive suitable marginal accuracy for restorative reconstruction.
